# Diurnal timing of physical activity and risk of colorectal cancer in the UK Biobank

**DOI:** 10.1186/s12916-024-03632-4

**Published:** 2024-09-18

**Authors:** Michael J. Stein, Hansjörg Baurecht, Patricia Bohmann, Béatrice Fervers, Emma Fontvieille, Heinz Freisling, Christine M. Friedenreich, Julian Konzok, Laia Peruchet-Noray, Anja M. Sedlmeier, Michael F. Leitzmann, Andrea Weber

**Affiliations:** 1 Department of Epidemiology and Preventive Medicine, University of Regensburg, Franz-Josef-Strauß-Allee 11, Regensburg, 93053 Germany; 2 Département Prévention Cancer Environnement, Centre Léon Bérard, Lyon, France; 3 INSERM U1296 Radiations: Défense, Santé, Environnement, Lyon, France; 4 International Agency for Research On Cancer (IARC), Nutrition and Metabolism Branch, 25 Avenue Tony Garnier, CS90627, Lyon, 69366 France; 5 Department of Cancer Epidemiology and Prevention Research, Alberta Health Services, Calgary, AB Canada; 6 Departments of Oncology and Community Health Sciences, Cumming School of Medicine, University of Calgary, Calgary, AB Canada; 7 Department of Clinical Sciences, Faculty of Medicine, University of Barcelona, Barcelona, Spain; 8 Center for Translational Oncology, University Hospital Regensburg, Regensburg, 93053 Germany; 9 Bavarian Cancer Research Center (BZKF), Regensburg, 93053 Germany

**Keywords:** Physical activity patterns, Colorectal cancer, UK Biobank, Raw accelerometry

## Abstract

**Background:**

Physical activity reduces colorectal cancer risk, yet the diurnal timing of physical activity in colorectal cancer etiology remains unclear.

**Methods:**

This study used 24-h accelerometry time series from UK Biobank participants aged 42 to 79 years to derive circadian physical activity patterns using functional principal component analysis. Multivariable Cox proportional hazard models were used to examine associations with colorectal cancer risk.

**Results:**

Among 86,252 participants (56% women), 529 colorectal cancer cases occurred during a median 5.3-year follow-up. We identified four physical activity patterns that explained almost 100% of the data variability during the day. A pattern of continuous day-long activity was inversely associated with colorectal cancer risk (hazard ratio (HR) = 0.94, 95% confidence interval (CI) = 0.89–0.99). A second pattern of late-day activity was suggestively inversely related to risk (HR = 0.93, 95% CI = 0.85–1.02). A third pattern of early- plus late-day activity was associated with decreased risk (HR = 0.89, 95% CI = 0.80–0.99). A fourth pattern of mid-day plus night-time activity showed no relation (HR = 1.02, 95% CI = 0.88–1.19). Our results were consistent across various sensitivity analyses, including the restriction to never smokers, the exclusion of the first 2 years of follow-up, and the adjustment for shift work.

**Conclusions:**

A pattern of early- plus late-day activity is related to reduced colorectal cancer risk, beyond the benefits of overall activity. Further research is needed to confirm the role of activity timing in colorectal cancer prevention.

**Supplementary Information:**

The online version contains supplementary material available at 10.1186/s12916-024-03632-4.

## Background

The global prevalence of insufficient physical activity in 2016 was 28%, with a higher rate (37%) in high-income countries [1, 2]. There is substantial evidence of a dose–response relation between increasing levels of physical activity and decreasing incidence of at least 10 different cancers including colorectal cancer [3]. Whilst evidence has accumulated on the type, dose, and time periods in life when physical activity is associated with reduced cancer risk, the specific impact of its timing during the day is poorly understood.

Diurnal timing of exercise impacts muscle metabolism [4] and may influence cardiometabolic processes that play a role in carcinogenesis [3]. Recent studies have explored its association with various health outcomes, yielding diverse findings [5]. Mid-day or afternoon activity has been associated with lower blood glucose levels [6–8] and decreased mortality [9]. Evening activity has been linked to improved cardiometabolic health markers [10–12] and a lower body mass index (BMI) [13]. Morning activity has yielded inconsistent results with cardiovascular disease; one study suggested a decreased risk [14], while another indicated an increased risk [15]. The evidence for an association between time-of-day specific activity with cancer risk is sparse and inconsistent. One study reported decreased colorectal cancer risk with morning and afternoon activity [16], whereas other investigations found no relations of activity timing to risks of breast and prostate cancer [17] or cancer mortality [9].

Device-based measurement of physical activity, increasingly common in health research [16], facilitates the assessment of diurnal physical activity timing. However, available research using accelerometer-based assessments for activity timing in relation to cancer is very limited and has faced challenges such as the need for a priori assumptions in clustering algorithms [16] and the risk of overfitting with dataset-specific time windows [9].

To address those issues, we used functional principal component analysis (fPCA) to assess diurnal activity patterns and their relations to colorectal cancer. Given the limited evidence for the effects of diurnal timing of physical activity on cancer risk, we focused on a malignancy that is remarkably responsive to physical activity as a preventive measure [18], hypothesizing that potential associations are most likely to occur here. fPCA extends PCA to handle functions or curves as observations, efficiently reducing data complexity without pre-set assumptions. Our aim was to overcome previous methodologic limitations and identify specific times of day when physical activity is potentially most effective in preventing colorectal cancer.

## Methods

### Study population and data collection

The UK Biobank, a prospective cohort study, enrolled over 500,000 UK participants aged 40 to 69 years between 2006 and 2010. The study collected sociodemographic, lifestyle, and extensive phenotypic data, using touchscreen questionnaires, interviews, physical and functional measurements, and biomaterials collection. Ethical approval was obtained from the North West Multi-Centre Research Ethics Committee. All participants provided written informed consent [19].

### Physical activity assessment and patterns

In a subset of over 103,000 randomly selected participants, device-based physical activity was measured between 2013 and 2015 using an Axivity AX3 wrist-worn triaxial accelerometer (Newcastle Upon Tyne, UK). Participants were instructed to wear the accelerometer on their dominant wrist continuously for seven days from activation soon after receiving it, and then to return the device to the coordinating center. A UK Biobank expert group processed data to derive the Euclidean norm minus one (ENMO) from accelerometry data [20], a summary metric of bodily acceleration in milligravity units (m*g*), interpretable as physical activity volume. Inclusion criteria included data with good calibration and from at least 72 h, covering each hour of the day on multiple days. Those with daily ENMOs above the 99.9^th^ percentile were excluded, leaving 96,568 participants (Additional file 1: Supplement S1). These data formed a 96,568 × 24 matrix of average hourly acceleration.

### Functional principal component analysis

We estimated standardized residuals of the 24-h ENMO time series using linear regression adjusted for age, sex, and study region to address major confounding a priori. These residuals underwent fPCA to reduce data dimensionality while retaining between-person variation. Individuals’ fPC scores indicated how closely a participant’s activity data matched a specific pattern (eigenfunction) [21]. fPCA was implemented using principal analysis by conditional estimation (PACE) suitable for sparse longitudinal data [22]. Gaussian kernel smoothing with default bandwidth estimation (5% of the observed time range for the mean function; 10% for the covariance function) was applied. Robustness was assessed through sensitivity analyses using generalized cross-validation for bandwidth selection along with an alternative kernel smoothing method. The Epanechnikov kernel was chosen for its compactness and performance. The number of relevant components was determined using the elbow method, a > 95% variability threshold, and visual inspection of fPCs [23].

To identify activity patterns that encompass all movements captured by the accelerometer, including very low-intensity activity, we applied fPCA to time series accelerometry data. This method calculates multiple components, with each participant assigned a score for each component, indicating their alignment with respective patterns. Scores are either positive or negative, reflecting the degree to which a participant’s activity matches the periods when the fPC curve is positive or negative. A more extreme score signifies stronger adherence to that activity pattern, allowing for a comprehensive interpretation of individual activity behaviors in relation to these identified patterns.

### Cohort follow-up and ascertainment of cancer cases

Participants’ vital status was obtained through linkage with health care data and national death registries [24]. Follow-up began at the baseline accelerometry measurement (June 2013–December 2015) and ended at cancer diagnosis, complete follow-up (February 2020 for England/Wales, January 2021 for Scotland) [25], loss to follow-up, or death, whichever came first. We focused on colorectal cancer incidence given convincing evidence for its relation to physical activity [18]. Colorectal cancer was classified using International Classification of Diseases (ICD-10) codes C18, C19, and C20. Only the first primary cancers were considered.

### Covariates

Potential confounding covariates were identified using evidence-derived directed acyclic graphs and the disjunctive cause criterion [26] (Additional file 1: Supplement S2). Covariate details are given in Additional file 1: Supplement S3. Briefly, we stratified by sex, study region (England, Scotland, Wales), and age at accelerometry (10-year increments), and further adjusted for BMI (kg/m^2^), height (cm), smoking (pack years), alcohol use (grams per day [27]), self-reported sedentary behavior (hours) (as continuous variables), socio-economic status (Townsend index), education (College/University Degree; Higher National Diploma, A-level, other professional qualifications; General Certificate of Secondary Education, O-level; or none), diet (healthy diet score [28], 0–7 scale) (as categorical variables), hormone therapy among women, history of cardiometabolic disease, family history of colorectal cancer, and history of bowel cancer screening (as binary variables).

### Statistical analysis

Statistical analysis included 86,252 participants after excluding 10,316 with prevalent malignant cancers (except non-melanoma skin cancer, Additional file 1: Supplement S1).

To address missing covariate data (0.1% to 15%, Additional file 1: Supplement S4), multiple imputation using chained equations was applied (ten datasets with five iterations each). This imputation involved predictive mean matching for continuous variables, logistic regression for binary variables, polytomous logistic regression for nominal variables, and proportional odds models for ordinal variables. Convergence and plausibility of the imputation were assessed visually [29].

We conducted Cox proportional hazard regression with age as the underlying time metric [30] and mutual adjustment for the four fPCs modeled as continuous variables. We fitted three models: model 1 (sex, study region, age), model 2 (all covariates except BMI), and model 3 (model 2 plus BMI). We estimated hazard ratios (HRs) and corresponding 95% confidence intervals (CIs) for associations between fPC scores and cancer, comparing scores of + 1 (activity during hours with a positive fPC curve) and − 1 (activity during hours with a negative fPC curve) to a score of 0 (not fitting the fPC). For ease of interpretation, we reversed positive scores to consistently yield inverse associations. We estimated p-values using a Wald test and a 5% statistical significance level. Non-linearity was addressed using restricted cubic splines with four knots at the 0.05, 0.35, 0.65, and 0.95 quantiles. Departures from linearity were assessed by testing whether the coefficient of the second and third spline transformation equaled zero [30]. Proportional hazards assumptions were checked using Schoenfeld residuals, and none of the model assumptions was violated. Additionally, we examined Pearson and Spearman correlations between activity patterns and selected biomarkers, including glucose, glycated hemoglobin (HbA1c), high-density lipoprotein cholesterol, low-density lipoprotein cholesterol, triglycerides, estradiol, and insulin-like growth factor-1.

We conducted several sensitivity analyses to ensure the result robustness. These included disregarding the initial two follow-up years to address reverse causation, focusing on never-smokers to assess smoking-related confounding, analyzing colon and rectal cancer risk separately for anatomic site differences, examining potential collider bias by not adjusting for cardiometabolic disease, and exploring effect modification of the fPCA-cancer relations by all covariates to avoid missing subgroup associations. Additionally, we incorporated shift work in the model to investigate circadian rhythm disruptions. We also tested various fPCA hyperparameters (kernel smoother and bandwidths) and examined correlations with sleep, sedentary time, light, and moderate-to-vigorous activity proportions to evaluate activity pattern robustness.

All data processing and statistical analyses were performed using R 4.2.3 [31]. Specifically, fPCs were generated using the *fdapace* package [32], multiple imputation was conducted using the *mice* package [29], and Cox regression was performed using the *rms* package [33].

## Results

**Fig. 1:**
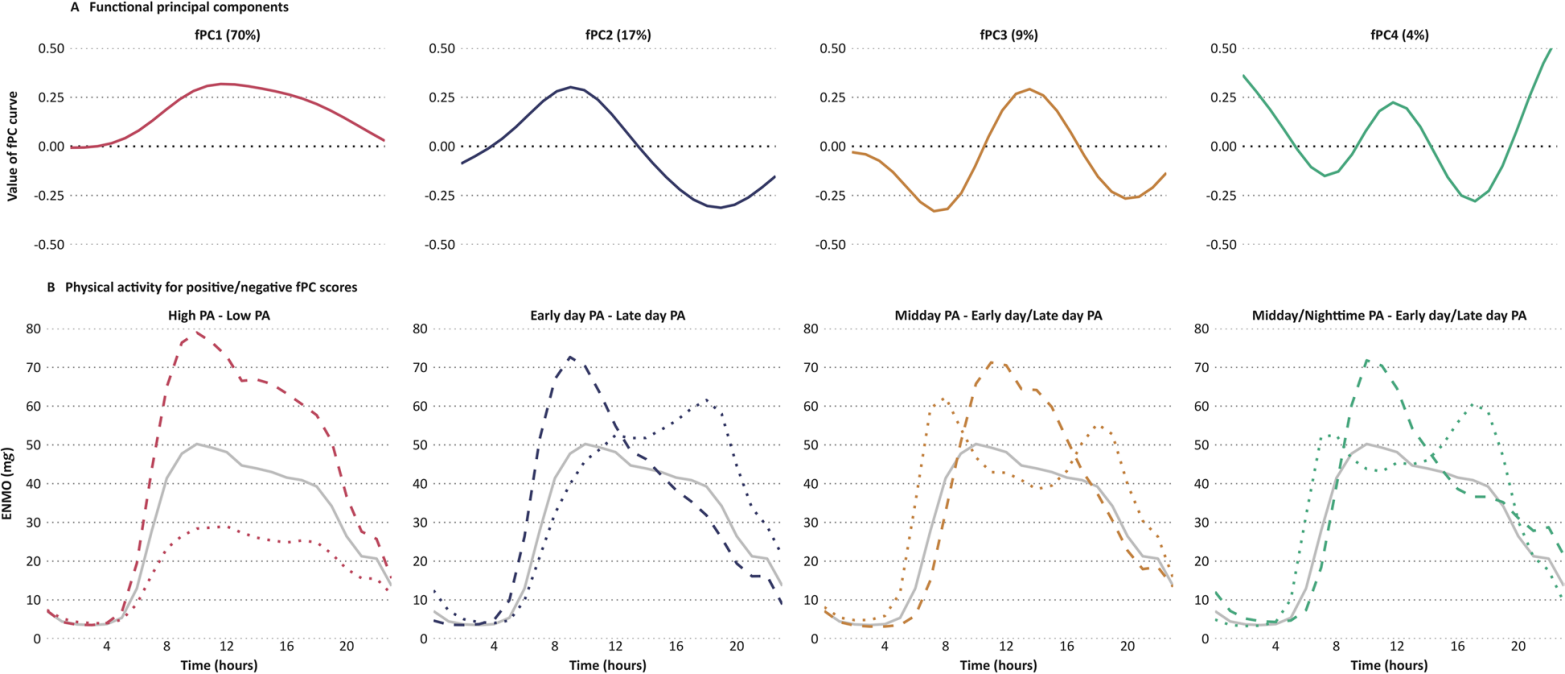
**A** Four distinct previously derived physical activity patterns from functional principal component analysis. **B** Average hourly physical activity (PA) for positive scores (dashed line; > 1 standard deviation above the mean score), negative scores (dotted line; < 1 standard deviation below the mean score), and population average (solid gray line), for each functional principal component

Among 86,252 participants (56% women) aged 61.5 years at accelerometry, 529 colorectal cancer cases were ascertained during 5.3 years of follow-up. We derived four fPCs explaining almost 100% of accelerometry data variability. The first pattern (fPC1, 70%) denoted day-long activity, fPC2 (17%) characterized late-day activity, fPC3 (9%) signified early- plus late-day activity, and fPC4 (4%) represented mid-day plus night-time activity (Fig. 1A). Further details on how fPC scores relate to activity over time are shown in Fig. 1B and Additional file 1: Supplement S5.

Table 1 presents baseline population characteristics by fPC score quartiles. Participants with higher scores on fPC1 (day-long activity) tended to show a healthier lifestyle, characterized by greater overall acceleration, lower BMI, reduced smoking habits, a healthier diet, less sedentary behavior, and lower prevalence of cardiometabolic diseases, relative to participants with lower scores. Those with fPC2 scores signifying late-day activity had a slightly healthier profile compared to those with early-day activity. This was due to higher overall acceleration levels, slightly lower BMI, decreased smoking, and sedentary habits, but higher alcohol consumption. Individuals with fPC3 scores representing early- plus late-day activity had a distinctly healthier lifestyle, marked by higher overall acceleration, decreased alcohol drinking and smoking habits, and lower sedentary behavior. Study participants with higher fPC4 scores (mid-day plus night-time activity) showed a slightly less healthy lifestyle with respect to increased tobacco use and sedentary lifestyle compared to individuals with lower fPC4 scores.

**Table 1:** Baseline characteristics of UK Biobank participants in 2006–2010 (accelerometry in 2013–2015) by the first and fourth age-standardized quantile of fPC scores

Characteristics	fPC1		fPC2		fPC3		fPC4	
	**Q1**	**Q4**	**Q1**	**Q4**	**Q1**	**Q4**	**Q1**	**Q4**
Sex	*N* (%)/ mean (sd)	*N* (%)/ mean (sd)	*N* (%)/ mean (sd)	*N* (%)/ mean (sd)	*N* (%)/ mean (sd)	*N* (%)/ mean (sd)	*N* (%)/ mean (sd)	*N* (%)/ mean (sd)
* Women*	10,581 (49%)	12,484 (58%)	12,058 (56%)	11,093 (51%)	11,222 (52%)	11,764 (55%)	10,971 (51%)	12,063 (56%)
* Men*	10,983 (51%)	9079 (42%)	9506 (44%)	10,470 (49%)	10,342 (48%)	9799 (45%)	10,593 (49%)	9500 (44%)
Age accelerometry, years	61.5 (7.9)	61.4 (7.8)	61.2 (7.8)	61.7 (7.9)	61.3 (7.9)	61.5 (7.7)	61.0 (7.8)	61.8 (7.8)
Average overall acceleration, m*g*	19.5 (3.6)	38.3 (6.6)	30.7 (8.2)	29.5 (8.6)	30.7 (8.7)	29.4 (8.0)	29.6 (8.6)	30.3 (8.2)
Light physical activity, min/week	1521.2 (469.9)	2646.3 (678.5)	2300.3 (708.2)	2102.0 (676.0)	2210.3 (733.9)	2145.0 (633.0)	2148.7 (697.5)	2201.9 (693.3)
Moderate-to-vigorous physical activity, min/week	149.1 (130.0)	480.0 (301.9)	323.0 (253.4)	349.7 (269.7)	320.2 (245.7)	361.2 (280.1)	334.6 (259.3)	334.3 (260.3)
Height, cm	170.2 (9.3)	168.6 (8.9)	169.2 (9.1)	169.5 (9.1)	169.7 (9.2)	169.2 (9.0)	170.0 (9.2)	168.9 (9.0)
Body mass index, kg/m^2^	28.5 (5.3)	25.2 (3.6)	26.2 (4.3)	26.7 (4.4)	26.3 (4.4)	26.4 (4.1)	26.4 (4.3)	26.4 (4.3)
Townsend Index of Deprivation	− 1.4 (3.0)	− 1.9 (2.7)	− 1.6 (2.9)	− 1.7 (2.8)	− 1.5 (2.9)	− 1.9 (2.7)	− 1.8 (2.8)	− 1.6 (2.9)
Education level								
* Highest*	9271 (43%)	9116 (43%)	10,265 (48%)	8070 (38%)	10,303 (48%)	8311 (39%)	9489 (44%)	9168 (43%)
* Intermediate*	5159 (24%)	4941 (23%)	5025 (24%)	5081 (24%)	4938 (23%)	5155 (24%)	5149 (24%)	4996 (23%)
* Lowest*	5040 (24%)	5512 (26%)	4715 (22%)	5827 (27%)	4708 (22%)	5832 (27%)	5217 (24%)	5215 (24%)
* None of the above*	1847 (8.7%)	1802 (8.4%)	1334 (6.3%)	2379 (11%)	1411 (6.6%)	2057 (9.6%)	1512 (7.1%)	1960 (9.2%)
Alcohol intake, g/day	16.5 (18.5)	16.4 (15.9)	17.3 (17.6)	16.4 (17.2)	15.7 (16.6)	17.4 (17.2)	16.7 (17.0)	16.6 (17.1)
Pack years, years	8.5 (16.0)	5.2 (11.1)	6.4 (13.1)	6.9 (13.6)	6.0 (12.9)	6.7 (12.9)	6.2 (12.7)	6.6 (13.1)
Healthy diet score	3.5 (1.3)	3.9 (1.3)	3.7 (1.3)	3.7 (1.3)	3.8 (1.3)	3.7 (1.3)	3.7 (1.3)	3.7 (1.3)
Sedentary behavior, h	4.9 (2.7)	3.8 (2.3)	4.1 (2.5)	4.4 (2.4)	4.0 (2.5)	4.4 (2.4)	4.1 (2.5)	4.3 (2.5)
Cardiometabolic disease								
* No*	17,538 (81%)	19,972 (93%)	19,301 (90%)	19,092 (89%)	19,156 (89%)	19,285 (89%)	19,232 (89%)	19,214 (89%)
* Yes*	4026 (19%)	1591 (7.4%)	2263 (10%)	2471 (11%)	2408 (11%)	2278 (11%)	2332 (11%)	2349 (11%)

m*g *milligravity unit, *sd *standard deviation

Highest education: college or university; intermediate: A/AS, NVQ/HND/HNC or equivalent, other qualifications; lowest: O/GCSEs, CSEs or equivalent

We examined correlations between the four fPCs and circulating biomarkers and noted a weak positive correlation between day-long activity (fPC1) and high-density lipoprotein cholesterol (men: *r* = 0.21, women: *r* = 0.17), as well as a weak negative correlation with triglycerides (men: *r* =  − 0.13, women: *r* =  − 0.15) and a weak negative correlation with HbA1c among men (*r* =  − 0.13). There were no meaningful correlations with other biomarkers or with other fPCs (Additional file 1: Supplement S6).

### Colorectal cancer risk

Increasing level of day-long activity (i.e., a 1-unit increase in the fPC1 score) showed an inverse association with colorectal cancer risk in the minimally adjusted model (HR = 0.92, 95% CI = 0.88–0.97). Multivariable adjustment had little impact on the relation (HR = 0.93, 95% CI = 0.88–0.98), and further adjustment for BMI yielded a similar result (HR = 0.94, 95% CI = 0.89–0.99). Ascending level of late-day activity (i.e., a 1-unit decrease in the fPC2 score) exhibited an inverse, but statistically non-significant relation with colorectal cancer, regardless of the degree of adjustment (HR = 0.93, 95% CI = 0.85–1.02). Conversely, an increasing level of early- plus late-day activity, instead of mid-day activity (i.e., a 1-unit decrease in the fPC3 score) was inversely associated with colorectal cancer in the minimally adjusted model (HR = 0.89, 95% CI = 0.80–0.99), and in the multivariable-models with and without additional adjustment for BMI (HR = 0.89, 95% CI = 0.80–0.99). Lastly, mid-day plus night-time activity (i.e., a 1-unit increase in the fPC4 score) showed no relation to colorectal cancer (Table 2).

**Table 2:** Colorectal cancer risk (hazard ratios and 95% confidence intervals) for the four fPCs for models 1–3; *N* = 86,252, cases = 529

fPC	Activity timing	Model 1	Model 2	Model 3
fPC1	Higher overall vs. lower overall	0.92, 0.88–0.97	0.93, 0.88–0.98	0.94, 0.89–0.99
	*Non-linear p*	0.059	0.077	0.095
fPC2	Late-day vs. early-day	0.93, 0.85–1.02	0.93, 0.85–1.02	0.93, 0.85–1.02
	*Non-linear p*	0.647	0.573	0.569
fPC3	Early/late-day vs. mid-day	0.89, 0.80–0.99	0.89, 0.80–0.99	0.89, 0.80–0.99
	*Non-linear p*	0.514	0.489	0.486
fPC4	Mid-day/night vs. early/late-day	1.03, 0.88–1.20	1.02, 0.88–1.19	1.02, 0.88–1.19
	*Non-linear p*	0.275	0.285	0.292

Model 1: Four fPCs and stratified by sex, age, study region; model 2: model 1 + cardiometabolic disease, height, smoking, alcohol intake, socio-economic status, education, sedentary behavior, healthy diet score, hormone replacement therapy, family history of colorectal cancer, and bowel cancer screening; model 3: model 2 + body mass index

*fPC* functional principal component

Note: Non-linear *p*-values were estimated by testing whether the coefficient of the second and third spline transformation equaled zero. To ease interpretation, hazard ratios for fPC1 and fPC4 are presented for a score comparison of + 1 vs. 0, while for fPC2 and fPC3, they are for a score of − 1 vs. 0

### Sensitivity and interaction analyses

Separately considering colon (349 cases) and rectal cancers (180 cases), late-day activity (fPC2) showed no relation to colon cancer (HR = 0.96, 95% CI = 0.86–1.08), but was suggestively inversely associated with rectal cancer (HR = 0.88, 95% CI = 0.76–1.02) (model 3: *p* for difference = 0.369, Table 3). After excluding the first two years of follow-up, the association between day-long activity (fPC1) and colorectal cancer slightly weakened (HR for fPC1 = 0.94, 95% CI = 0.88–1.00), while the relation with early- plus late-day activity strengthened (HR for fPC3 = 0.83, 0.72–0.96, Additional file 1: Supplement S7). Limiting the analysis to never-smokers did not materially influence the inverse association between early- plus late-day activity (fPC3) and colorectal cancer, but the CIs were wider, now including the null value (HR = 0.87, 95% CI = 0.74–1.02, Additional file 1: Supplement S8). Excluding the term for cardiometabolic disease history from our models had no impact (HR for fPC1 = 0.94, 95% CI = 0.89–0.99; HR for fPC3 = 0.89, 95% CI = 0.80–0.99, Additional file 1: Supplement S9). Additionally accounting for shift work status also had no effect (Additional file 1: Supplement S10).

**Table 3:** Colon and rectal cancer risk (hazard ratios and 95% confidence intervals) for the four fPCs for models 1–3

**fPC**	**Activity timing**	**Model**	**Colon** Cases = 349	**Rectum** Cases = 180	***P* for difference**
fPC1	Higher overall vs lower overall	Model 1	0.89, 0.84–0.95	0.97, 0.90–1.06	0.073
		Model 2	0.91, 0.85–0.96	0.97, 0.90–1.05	0.181
		Model 3	0.92, 0.86–0.98	0.97, 0.90–1.06	0.302
fPC2	Late-day vs early-day	Model 1	0.96, 0.86–1.08	0.89, 0.77–1.03	0.398
		Model 2	0.96, 0.85–1.08	0.88, 0.76–1.02	0.381
		Model 3	0.96, 0.86–1.08	0.88, 0.76–1.02	0.369
fPC3	Early/late-day vs mid-day	Model 1	0.88, 0.76–1.00	0.92, 0.77–1.10	0.654
		Model 2	0.88, 0.76–1.01	0.91, 0.77–1.10	0.735
		Model 3	0.88, 0.77–1.01	0.91, 0.77–1.09	0.746
fPC4	Mid-day/night vs early/late-day	Model 1	1.03, 0.85–1.25	1.03, 0.80–1.32	0.994
		Model 2	1.02, 0.84–1.24	1.03, 0.80–1.32	0.950
		Model 3	1.02, 0.84–1.24	1.03, 0.80–1.32	0.953

Model 1: Four fPCs and stratified by sex, age, study region; model 2: model 1 + cardiometabolic disease, height, smoking, alcohol intake, socio-economic status, education, sedentary behavior, healthy diet score, hormone replacement therapy, family history of colorectal cancer, and bowel cancer screening; model 3: model 2 + body mass index

*fPC* functional principal component

Note: To ease interpretation, hazard ratios for fPC1 and fPC4 are presented for a score comparison of + 1 vs. 0, while for fPC2 and fPC3, they are for a score of − 1 vs. 0

Interaction analyses revealed that day-long activity (fPC1) was inversely associated with colorectal cancer mainly in individuals who fell into the third quartile of sedentary behavior (HR = 0.87, 95% CI = 0.77–0.96), but not in those in the bottom quartile (HR = 0.98, 95% CI = 0.90–1.06; *p* for interaction = 0.036). Among women, day-long activity was inversely related to colorectal cancer in never-users of hormone therapy (HR = 0.88, 95% CI = 0.79–0.98), with no association in users (HR = 1.06, 95% CI = 0.95–1.19, *p* for interaction = 0.049) (Table 4, Additional file 1: Supplement S11).

**Table 4:** Association of fPC1 with colorectal cancer risk by subgroups of sedentary behavior and hormone therapy

Sub analysis	*N*/cases	Hazard ratio (95% confidence interval)	*P* for interaction
fPC1 and sedentary behavior			0.036
* Q1 (*< *3 h)*	35,395/194	0.98, 0.90–1.06	
* Q2 (3–4 h)*	16,246/106	0.94, 0.84–1.06	
* Q3 (4–6 h)*	21,374/132	0.87, 0.77–0.96	
* Q4 (*> *6 h)*	13,170/97	0.92, 0.81–1.05	
fPC1 and hormone therapy			0.049
* No*	30,709/125	0.88, 0.79–0.98	
* Yes*	17,015/106	1.06, 0.95–1.19	

*fPC *functional principal component

Our fPCs were robust for variation in the bandwidths of the kernel smoother. When using an Epanechnikov kernel for smoothing, the explained variability was smaller for fPC1 (~ 18% decrease) and six components were necessary to reach the 95% threshold (Additional file 1: Supplement S12). However, the shapes of the first four fPCs were similar (Additional file 1: Supplement S13). fPCs were weakly to moderately correlated with accelerometer-derived physical activity intensities (Additional file 1: Supplement S14).

## Discussion

Our primary finding was the identification of a two-peak pattern that was associated with reduced colorectal cancer risk, beyond the benefits of overall physical activity. That pattern of early- plus late-day activity (fPC3) was characterized by two distinct activity peaks: one in the morning at around 8AM and another in the afternoon at around 6PM. We also identified a pattern of late-day activity (fPC2), marked by a single peak in activity at approximately 6PM. However, that pattern was associated with a less pronounced decrease in colorectal cancer risk compared to the double peak pattern and did not reach statistical significance. The more pronounced benefit of the double peak activity pattern, as opposed to the single peak pattern, could be partially attributable to the advantage of distributing activities throughout both the morning and the afternoon, providing more comprehensive coverage of active time during the day.

Physical activity and colorectal cancer share a dose–response relationship, with additional benefits beyond the recommended levels of physical activity [34]. The evidence is strong enough to support a convincing causal association [18]. To our knowledge, this study is the first to use accelerometer data to examine fPCA-derived circadian physical activity patterns and their relationship to colorectal cancer risk.

Existing literature on timing of physical activity in relation to cancer is limited and includes only three studies. The first, a case–control study by Weitzer et al. [17], used interviewer-assessed physical activity data. It reported statistically non-significant decreased odds ratios of breast and prostate cancer with early morning activity but found no relation with mid-day or afternoon activity. The second, a prospective study by Feng et al., used UK Biobank accelerometer data and categorized physical activity into predetermined time intervals but observed no association with cancer mortality [9]. The third, a study by Bai et al., also used UK Biobank accelerometer data and it utilized k-means cluster analysis to discern circadian physical activity patterns. That study noted a reduced risk of colorectal cancer associated with activity in both the morning and afternoon [16], a finding that is consistent with our study results, supporting a potential benefit of a two-peak activity pattern in reducing colorectal cancer risk. We expand on this knowledge by using fPCA, which is not based on a priori assumptions, in contrast to the clustering method employed by Bai et al. Secondly, while Bai et al. compared the discrete membership of the double-peaked cluster with that of a consistently inactive subgroup—a comparison that may possibly increase the likelihood of statistical significance, we avoided this approach to maintain a more conservative analysis. Thirdly, we rigorously accounted for potential confounding variables through causal inference methods, providing a more robust analysis than Bai et al. Fourthly, unlike Bai et al., we included only participants with valid accelerometer data, ensuring the validity of our physical activity measurements. Finally, our study incorporated a more extensive range of sensitivity and interaction analyses than Bai et al., offering a more comprehensive understanding of the data. Considering the potential benefits of two-peak diurnal activity, it is important to note that the evidence on physical activity and health emphasizes that every move counts, regardless of intensity or duration [35]. In this regard, our findings suggest that instead of accumulating activity once a day, it may be better to engage in daily activity throughout the day.

In supplementary analyses, we observed that a day-long activity pattern (fPC1) was inversely associated with colorectal cancer risk particularly among individuals with higher levels of sedentary behavior. One possible explanation for this finding is that the apparent protective effect of physical activity becomes more pronounced when contrasted with prolonged periods of sedentary behavior. Previous investigations have not found that the physical activity and colorectal cancer relation is modified by sedentary behavior [36–39]. Of note, the general physical activity levels of the present cohort were relatively high. Therefore, individuals with less sedentary habits may exhibit an optimized cancer risk profile, leaving less room for further benefits from increased physical activity. Moreover, day-long activity (fPC1) seemed to be less relevant for rectal cancer, possibly due to less power in the rectal compared to the colon cancer analysis. However, the risk estimates for late-day activity (fPC2) were suggestively stronger for rectal than for colon cancer, hinting at a yet unknown link between physical activity and rectal cancer. Given the distinct relationships between the diurnal timing of physical activity and colon and rectal cancers, along with the varying carcinogenic processes for each type [40], future research is warranted to investigate diurnal timing of physical activity in relation to colorectal cancer according to anatomic subsite.

Among women, the day-long activity pattern was related to decreased colorectal cancer risk exclusively in those not using hormone therapy. In contrast, no association was observed in users of hormone therapy. Such interaction has been previously reported [41, 42]. Because estrogen is inversely linked to colorectal cancer [43], the observation of a risk gradient in the physical activity and colorectal cancer relation according to menopausal hormone therapy usage implies that physical activity may be associated with reduced colorectal cancer risk in part through a mechanism involving estrogen.

The biologic mechanisms underlying how the timing of daily activity affects cancer risk remain elusive. Animal studies show that the circadian clock regulates metabolic responses to exercise, and that the timing of exercise plays a pivotal role in enhancing the positive effects of exercise on metabolic pathways and energy balance [44, 45], which, in turn, is associated with cancer risk reduction [46]. Additionally, human skeletal muscle oxidative metabolism, influenced significantly by exercise, follows a circadian pattern, with peak strength and mitochondrial function occurring in the late afternoon [47, 48].

The primary etiologic pathway linking activity timing to colorectal cancer likely involves insulin resistance, a well-established colon cancer risk factor [49]. However, studies examining activity timing in relation to insulin resistance have yielded varying results. Some indicate that higher physical activity in the morning is associated with improved insulin resistance [13] or less incidence of obesity [50], while others suggest that afternoon or evening activity is related to improved insulin resistance [12] or greater reduction in fat mass [51]. Intervention studies consistently show that engaging in exercise in the afternoon or evening more strongly reduces blood glucose, insulin, and triglyceride levels, compared to other times of the day [6–8, 11], suggesting that late-day activity is the most probable protective factor.

Another biologic mechanism whereby the timing of physical activity may impact colorectal cancer risk is by decreasing chronic low-grade inflammation, a known contributor to carcinogenesis [3]. Inflammatory cytokines follow the circadian rhythm [52], and engaging in physical activity at specific times of the day is associated with reduced systemic inflammation [16].

Melatonin, which plays a significant role in both the circadian rhythm and in carcinogenesis [53], is influenced by rest-activity chronotypes. Nonetheless, the interaction between activity timing and melatonin secretion remains unclear [54].

On a methodological note, different data-driven methods to assess device-based timing of physical activity reveal fairly consistent physical activity time periods. Using clustering algorithms, a double peak pattern was identified [16] that was comparable to our fPC3, and others found a “late morning” pattern with a physical activity trajectory comparable to our early-day pattern (fPC2) [14]. In contrast, our fPCA avoids constraining individuals into an a priori defined number of discrete cluster and provides an understanding of the significance of each pattern by quantifying the amount of variance explained.

### Strengths and limitations

The primary strength of our study lies in its novel exploration of diurnal activity timing in relation to colorectal cancer using fPCA. This method is free from pre-set assumptions about data structure, and it efficiently reduces data complexity and captures essential variation while maintaining the continuous nature of the data, rendering it ideal for understanding nuanced trends in time-series of raw accelerometry data. Capturing the entire range of acceleration signals provided us with a detailed perspective on overall activity timing. Another significant asset of our study is its large sample size, allowing us to perform a wide range of informative sub-analyses, confirming the robustness of our findings.

A limitation is our focus on hourly acceleration averages without distinguishing activity types or intensities, potentially masking certain aspects affecting colorectal cancer risk, such as the benefits of short bursts of vigorous activity [55]. The accelerometry data lacked contextual details, limiting insights into how different environments in which activity occurred could influence the impact of physical activity on colorectal cancer. Additionally, we did not examine whether chronotype or sleep patterns modified the association between activity timing and colorectal cancer. Case numbers were relatively low, especially in subgroup analyses, potentially masking true effects. UK Biobank is susceptible to selection bias [56] and the accelerometer subpopulation studied may exhibit healthy volunteer bias given the relatively high levels of activity [57, 58]. Finally, translating the fPCA findings into public health messages is challenging given the complexity of these analytic approaches. However, our results support physical activity recommendations that “every move counts.”

## Conclusions

This study fills a crucial gap in our understanding of the role of physical activity in cancer prevention by contributing valuable data to the sparse literature on diurnal activity patterns and colorectal cancer risk. Leveraging raw accelerometer data and advanced statistical techniques, we uncovered a distinct pattern of activity during early and late parts of the day, which was associated with reduced risk of colorectal cancer, independent of overall activity. Should this finding be substantiated, physical activity timing could emerge as an innovative approach to prevent colorectal cancer. As such, the identification of specific times of the day when physical activity is most beneficial bears potential to shape cancer prevention programs. Nevertheless, further research is needed to corroborate the role of activity timing in cancer prevention.

## Supplementary Information

Additional file 1: Supplement S1. Flowchart for inclusion and exclusion of participants. Supplement S2. Directed acyclic graph. Supplement S3. Covariates for Cox regression. Supplement S4. Missing information for covariates by fPC score quantiles. Supplement S5. Description of the physical activity patterns. Supplement S6. Correlation coefficients between fPCs and blood biomarkers. Supplement S7. Cox model results after exclusion of the first two years of follow-up. Supplement S8. Cox model results after restricting the analysis to never smokers. Supplement S9. Cox model results without adjustment for cardiometabolic disease status. Supplement S10. Cox model results with adjustment for shift work status. Supplement S11. Interaction terms for fPCs and covariates. Supplement S12. Sensitivity fPCA with different bandwidth estimations and kernel smoothers. Supplement S13. First four fPCs (A) and positive and negative scorers (B) when using an Epanechnikov kernel. Supplement S14. Correlation coefficients for fPCs and derived accelerometry.

## Data Availability

UK Biobank is an open access resource. Bona fide researchers can apply to use the UK Biobank dataset by registering and applying at http://ukbiobank.ac.uk/register-apply/.

## Abbreviations

BMI: Body mass index
CI: Confidence interval
ENMO: Euclidean norm minus one
fPCA: Functional principal component analysis
fPC: Functional principal component
HR: Hazard ratio
ICD: International Classification of Diseases
m*g*: Milligravity unit
PACE: Principal analysis by conditional estimation
