# Supplementary material for: Diurnal timing of physical activity and risk of colorectal cancer in the UK Biobank
Source: BMC Med. 2024 Sep 18;22:399. doi: 10.1186/s12916-024-03632-4 (PMC11409794; doi:10.1186/s12916-024-03632-4)
Supplement: Supplementary file 1 — Additional file 1: Supplement S1. Flowchart for inclusion and exclusion of participants. Supplement S2. Directed acyclic graph. Supplement S3. Covariates for Cox regression. Supplement S4. Missing information for covariates by fPC score quantiles. Supplement S5. Description of the physical activity patterns. Supplement S6. Correlation coefficients between fPCs and blood biomarkers. Supplement S7. Cox model results after exclusion of the first two years of follow-up. Supplement S8. Cox model results after restricting the analysis to never smokers. Supplement S9. Cox model results without adjustment for cardiometabolic disease status. Supplement S10. Cox model results with adjustment for shift work status. Supplement S11. Interaction terms for fPCs and covariates. Supplement S12. Sensitivity fPCA with different bandwidth estimations and kernel smoothers. Supplement S13. First four fPCs (A) and positive and negative scorers (B) when using an Epanechnikov kernel. Supplement S14. Correlation coefficients for fPCs and derived accelerometry. [file 12916_2024_3632_MOESM1_ESM.docx]

Diurnal timing of physical activity and risk of colorectal cancer in the UK Biobank

Supplementary material

Michael J. Stein, Hansjörg Baurecht, Patricia Bohmann, Béatrice Fervers, Emma Fontvieille, Heinz Freisling, Christine M. Friedenreich, Julian Konzok, Laia Peruchet-Noray, Anja M. Sedlmeier, Michael F. Leitzmann, Andrea Weber

**Corresponding author:** Michael J. Stein, Tel.: +49 941 944 521 6, Mail: michael.stein@ukr.de, Department of Epidemiology and Preventive Medicine, University of Regensburg, Regensburg, Germany

[S1. Flowchart for inclusion and exclusion of participants](#_Toc172034037)

[S2. Directed acyclic graph](#_Toc172034038)

[S3. Covariates for Cox regression](#_Toc172034039)

[S4. Missing information for covariates by fPC score quantiles](#_Toc172034040)

[S5. Description of the physical activity patterns](#_Toc172034041)

[S6. Correlation coefficients between fPCs and blood biomarkers](#_Toc172034042)

[S7. Cox model results after exclusion of the first two years of follow-up](#_Toc172034043)

[S8. Cox model results after restricting the analysis to never smokers](#_Toc172034044)

[S9. Cox model results without adjustment for cardiometabolic disease status](#_Toc172034045)

[S10. Cox model results with adjustment for shift work status](#_Toc172034046)

[S11. Interaction terms for fPCs and covariates](#_Toc172034047)

[S12. Sensitivity fPCA with different bandwidth estimations and kernel smoothers](#_Toc172034048)

[S13. First four fPCs (A) and positive and negative scorers (B) when using an Epanechnikov kernel](#_Toc172034049)

[S14. Correlation coefficients for fPCs and derived accelerometry](#_Toc172034050)

# Flowchart for inclusion and exclusion of participants

#
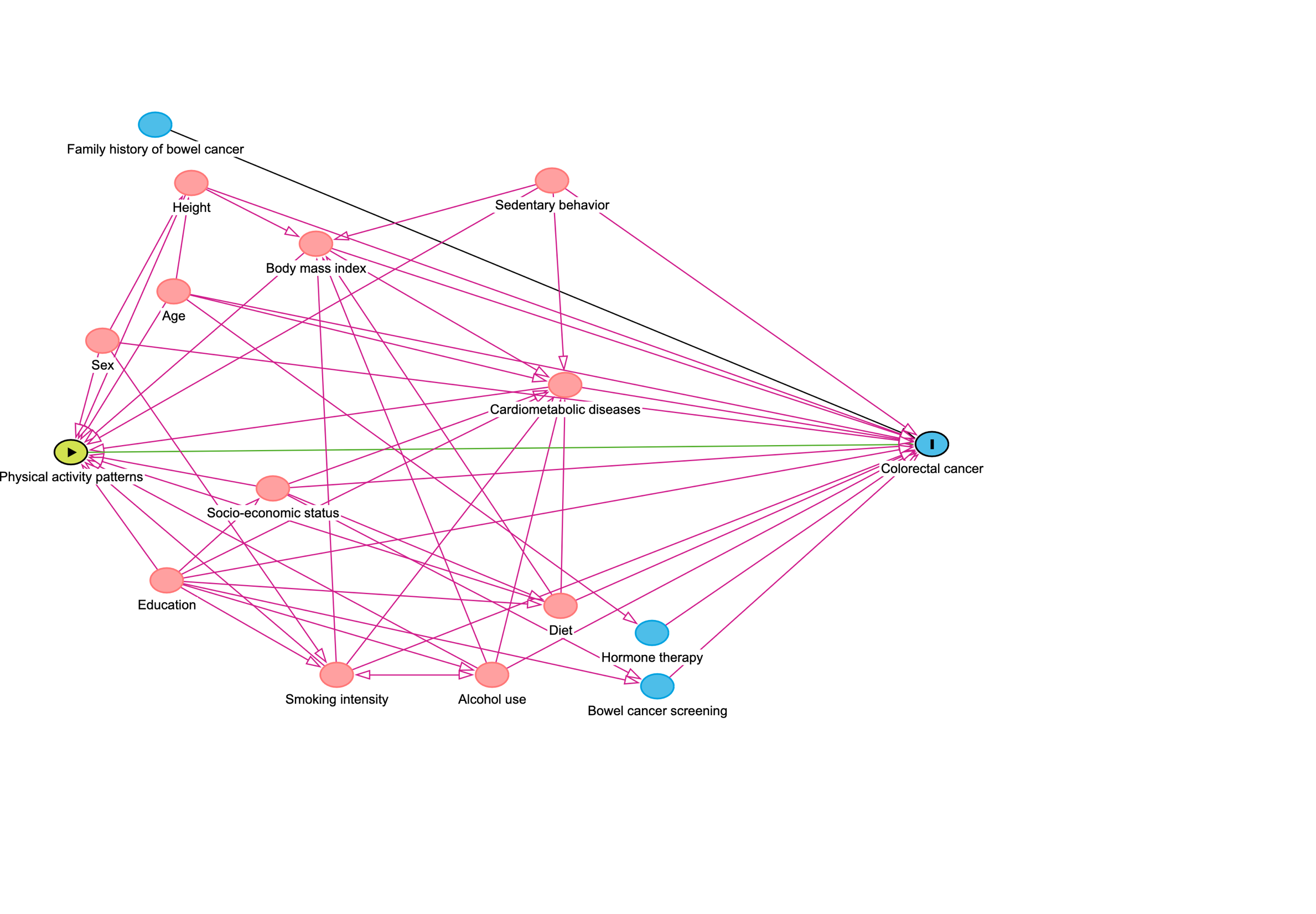
Directed acyclic graph

Hormone therapy, bowel cancer screening, and family history of bowel cancer are only causes of the outcome, hence, not confounding variables, but adjusting for such variables tends to increase the power of statistical tests [1].

[1] VanderWeele TJ, Shpitser I. A New Criterion for Confounder Selection. Biometrics; 2011;67(4):1406-13. https://doi.org/10.1111/j.1541-0420.2011.01619.x

# Covariates for Cox regression

| **Information on confounding variables.** | | | |
| --- | --- | --- | --- |
| **Covariate** | **UK Biobank – variable identification number** | **Calculation** | **Further Comments** |
| Alcohol use | ID 1558: Alcohol intake frequency  ID 1558: Alcohol intake frequency.  ID 1568: Average weekly red wine intake  ID 1578: Average weekly champagne plus white wine intake  ID 1588: weekly beer plus cider intake  ID 1598: Average weekly spirits intake  ID 1608: Average weekly fortified wine intake  ID 1618: Alcohol usually taken with meals  ID 1628: Alcohol intake versus 10 years previously  ID 3731: Former alcohol drinker  ID 4407: Average monthly red wine intake  ID 4418: Average monthly champagne plus white wine intake  ID 4429: Average monthly beer plus cider intake  ID 4440: Average monthly spirits intake  ID 4451: Average monthly fortified wine intake  ID 4462: Average monthly intake of other alcoholic drinks  ID 5364: Average weekly intake of other alcoholic drinks  ID 20117: Alcohol drinker status | Estimating daily alcohol consumption based on Bradbury et al. (1). We considered that a pint of beer contained 20 g and all other drinks 10 g of alcohol and summed the total weekly and monthly alcohol consumption accordingly. To obtain an estimated daily total, we divided the weekly consumption by 7 (or the monthly consumption by 30.4375). |  |
| Body mass index | ID 21001: Body mass index (BMI) | Weight (kg) divided by height (m) squared | Value not present if either weight or height were not available |
| Cardiometabolic diseases | ID 2443: Diabetes diagnosed by doctor  ID 6150: Vascular/heart problems diagnosed by doctor  ID 41202: Diagnoses – main ICD10  ID 41203: Diagnoses – main ICD9  ID 41204: Diagnoses – secondary ICD10  ID 41205: Diagnoses – secondary ICD9  ID 41262: Date of first in-patient diagnosis – main ICD10  ID 41263: Date of first in-patient diagnosis – main ICD9  ID 41270: Diagnoses – ICD10  ID 41271: Diagnoses – ICD9  ID 41280: Date of first in-patient diagnosis – ICD10  ID 41281: Date of first in-patient diagnosis – ICD9 | **Prevalent cardiovascular diseases:**  The following baseline self-reported diseases were used:   - 1 = Heart attack - 2 = Angina - 3 = Stroke   The following diseases and their ICD codes were defined as cardiovascular diseases:   - Angina pectoris: ICD 10: I20.0 - I20.9 & ICD9: 4139 - Acute myocardial infarction: ICD 10: I21.0 – I21.9 & ICD9: 4109 - Other acute ischemic heart diseases: ICD10: I24.0 – I24.9 & ICD9: 4119 - Chronic ischemic heart diseases: ICD10: I25.0 – I25.9 & ICD9: 4140, 4148, 4149 - Atrial fibrillation: ICD10: I48.0-I48.9 & ICD9: 4273 - Other cardiac arrhythmias: ICD10: I49.0 – I 49.9 & ICD9: 4270-4279 - Heart failure: ICD10: I50.0 – I50.9 & ICD9: 4280, 4281 - Cerebrovascular diseases (incl. stroke): ICD10: I60.0 – I60.9; I61.0 – I61.9; I62.0 – I62.9; I63.0 – I63.9; I64; I65.0 – I65.9; I66.0 – I66.9; I67.0 – I67.9; I68.0 – I68.9; I69.0 – I69.9 & ICD9: 4309, 4319, 4321, 4331, 4339, 4349, 4359, 4369, 4373, 4379, 4389 - Atherosclerosis: ICD10: I70.0 – I70.9 & ICD9: 4400, 4401, 4408, 4409 - Other peripheral vascular diseases: ICD10: I73.0 – I73.9 & ICD9: 4430, 4439   **Prevalent type 2 diabetes:**  Baseline self-reported diagnosis of diabetes was used as well as the following ICD codes:   - ICD10: E11.0 – E11.9   ICD9: 25000 |  |
| Diet | ID 1289: Cooked vegetable intake  ID 1299: Salad/raw vegetable intake  ID 1309: Fresh fruit intake  ID 1319: Dried fruit intake  ID 1329: Oily fish intake  ID 1339: Non-oily fish intake  ID 1349: Processed meat intake  ID 1359: Poultry intake  ID 1369: Beef intake  ID 1379: Lamb/Mutton intake  ID 1389: Pork intake  ID 1438: Bread intake  ID 1448: Bread type  ID 1458: Cereal intake  ID 1468: Cereal type | Building a healthy diet score based on Lourida et al. (2) ranging from 0 – 7 by giving one point per fulfilled nutritional category:   - Fruits: 3 servings/day - Vegetables: 3 servings/day - Fish: 2 servings/week - Processed meats: 1 serving/week - Unprocessed red meat: 1.5 servings/week - Whole grains: 3 servings/day - Refined grains: 1.5 servings/day | No inclusion of dairy intake (ID 1408, ID 1418, ID 1428)  Corrections:   - Vegetables & Fruit: <0 = 0; >5=5 |
| Education | ID 6138: Qualifications | Categorization in four groups:  1 = University or College degree  2 = A-level/professional qualification/HNC/NVQ  3 = 0-levels/CSE  4 = None |  |
| Family history of colorectal cancer | ID 20107: Illnesses of father  ID 20110: Illnesses of mother | 4 = Bowel cancer |  |
| Height | ID 50: Standing height | Measured in cm |  |
| History of bowel cancer screening | ID 2345: Ever had bowel cancer screening |  | Touchscreen question asked about screening test for bowel (colorectal) cancer, including blood and stool tests, colonoscopy and sigmoidoscopy |
| Hormone therapy | ID 2814: Ever used hormone-replacement therapy (HRT) | Baseline touchscreen question: “Have you ever used hormone replacement therapy (HRT)?” |  |
| Smoking | ID 20161: Pack years of smoking | Number of cigarettes per day / 20 * (Age stopped smoking - Age start smoking) |  |
| Sedentary behavior | ID 1070: Time spent watching TV  ID 1080: Time spent using computer  ID 1090: Time spent driving | Total sedentary behavior was calculated by adding up time spent watching television, time spent using computer and time spent driving.  Sedentary behavior while working was not included in our calculation. | The data was corrected by a truncation to 24h, if the sum was >24h.  Whenever there was a missing in either variable, the total sedentary behavior was considered as missing (n = 19,038) |
| Socio-economic status | ID 22189: Townsend deprivation index at recruitment | A composite score of employment, ownership of car and home, household overcrowding and postcode; higher values indicate a higher degree of deprivation. | The calculation happens prior to participating.  The values are rounded to 2 decimal places. |
| CSE: Certificate of Secondary Education; HNC: Higher National Certificate; MET: Metabolic equivalent of task; NVQ: National Vocational Qualification  **Literature**  [1] Bradbury KE, Murphy N, Key TJ. Diet and colorectal cancer in UK Biobank: a prospective study. International Journal of Epidemiology. 2019;49(1):246-58. doi:10.1093/ije/dyz064  [2] Lourida I, Hannon E, Littlejohns TJ, et al. Association of Lifestyle and Genetic Risk With Incidence of Dementia. JAMA. 2019;322(5):430-7. doi:10.1001/jama.2019.9879  [3] UK Biobank. Guidelines for data processing and analysis of IPAQ. Available at: https://biobank.ctsu.ox.ac.uk/crystal/refer.cgi?id=540 | | | |

# Missing information for covariates by fPC score quantiles

| **Characteristic** | **fPC1** | | **fPC2** | | **fPC3** | | **fPC4** | |
| --- | --- | --- | --- | --- | --- | --- | --- | --- |
|  | **Q1** | **Q4** | **Q1** | **Q4** | **Q1** | **Q4** | **Q1** | **Q4** |
| Sex | 0 | 0 | 0 | 0 | 0 | 0 | 0 | 0 |
| Age accelerometry, y | 0 | 0 | 0 | 0 | 0 | 0 | 0 | 0 |
| Average overall acceleration, m*g* | 0 | 0 | 0 | 0 | 0 | 0 | 0 | 0 |
| Light physical activity, min/wk | 0 | 0 | 0 | 0 | 0 | 0 | 0 | 0 |
| Moderate-to-vigorous physical activity, min/wk | 0 | 0 | 0 | 0 | 0 | 0 | 0 | 0 |
| Height, cm | 67 | 24 | 39 | 30 | 44 | 33 | 29 | 33 |
| Body mass index, kg/m^2^ | 93 | 32 | 51 | 41 | 57 | 42 | 40 | 41 |
| Townsend Index of Deprivation | 35 | 21 | 21 | 20 | 26 | 16 | 22 | 19 |
| Education level | 247 | 192 | 225 | 206 | 204 | 208 | 197 | 224 |
| Alcohol intake, g/d | 3,350 | 2,460 | 2,649 | 2,838 | 2,815 | 2,539 | 2,669 | 2,671 |
| Pack years, y | 2,956 | 3,395 | 3,395 | 3,148 | 3,309 | 3,258 | 3,304 | 3,231 |
| Healthy diet score | 10 | 12 | 14 | 13 | 11 | 10 | 12 | 9 |
| Sedentary behavior, h | 18 | 22 | 20 | 17 | 16 | 13 | 20 | 14 |
| Cardiometabolic disease | 0 | 0 | 0 | 0 | 0 | 0 | 0 | 0 |
| m*g*: milligravity unit | | | | | | | | |

# Description of the physical activity patterns

| **fPC** | **Definition** | **Score** | **Scoring effect** | **Approximate time period** |
| --- | --- | --- | --- | --- |
| fPC1 | Overall physical activity during the day | positive | higher overall physical activity | 06AM – 10PM |
|  |  | negative | lower overall physical activity | 06AM – 10PM |
| fPC2 | Contrast of early day versus late day physical activity | positive | early day physical activity | 04AM – 12PM |
|  |  | negative | late day physical activity | 12PM – 12AM |
| fPC3 | Contrast of midday versus early day and late day activity | positive | midday physical activity | 10AM – 04PM |
|  |  | negative | early day and late day physical activity | 04AM – 8AM and 04PM – 12AM |
| fPC4 | Contrast of midday and nighttime versus early day and late day activity | positive | midday and nighttime physical activity | 10AM – 04PM and 12AM – 04AM |
|  |  | negative | early day and late physical activity | 04AM – 08AM and 04PM – 08PM |
| Note: Since fPCs (functional principal components) are typically uncorrelated, the second, third, and fourth patterns are rather independent of the physical activity magnitude. This is, because the individual score for fPC1 – which summarizes overall physical activity – is uncorrelated to the scores for all other patterns. Therefore, a more extreme score means that the individual activity curve follows the pattern more strongly (in all directions). For example, a highly positive score on fPC2 (early day activity) does not necessarily correspond to high overall physical activity but instead, to elevated activity levels during early day hours as well as to non-elevated levels in the later day. | | | | |

# Correlation coefficients between fPCs and blood biomarkers

| **Men** |  |  |  |  |
| --- | --- | --- | --- | --- |
| **Biomarkers** | **fPC1** | **fPC2** | **fPC3** | **fPC4** |
| Glucose | -0.10 | 0.01 | 0.00 | 0.03 |
| HbA1c | -0.13 | 0.01 | 0.01 | 0.05 |
| HDL cholesterol | 0.21 | -0.01 | -0.03 | 0.00 |
| IGF-1 | 0.04 | -0.04 | -0.04 | -0.09 |
| LDL direct | 0.05 | 0.00 | 0.02 | -0.05 |
| Oestradiol | 0.00 | -0.04 | -0.05 | -0.05 |
| Triglycerides | -0.13 | 0.02 | 0.06 | 0.01 |

| **Women** |  |  |  |  |
| --- | --- | --- | --- | --- |
| **Biomarkers** | **fPC1** | **fPC2** | **fPC3** | **fPC4** |
| Glucose | -0.04 | -0.01 | 0.04 | 0.04 |
| HbA1c | -0.05 | 0.00 | 0.02 | 0.05 |
| HDL cholesterol | 0.17 | 0.01 | -0.01 | 0.04 |
| IGF-1 | 0.06 | -0.02 | -0.05 | -0.10 |
| LDL direct | -0.08 | 0.02 | 0.08 | 0.06 |
| Oestradiol | 0.01 | 0.01 | -0.04 | -0.04 |
| Triglycerides | -0.15 | 0.02 | 0.08 | 0.07 |

fPC: Functional principal component; HDL: High density lipoprotein; LDL: Low density lipoprotein

Correlation coefficients were derived using Pearson correlation, except for oestradiol and triglycerides, where the concentrations were not normally distributed; thus, Spearman correlations were calculated.

# Cox model results after exclusion of the first two years of follow-up

| **fPC** | **Activity timing** | **Model 1** | **Model 2** | **Model 3** |
| --- | --- | --- | --- | --- |
| fPC1 | Higher overall vs. lower overall | 0.90, 0.85-0.96 | 0.92, 0.87-0.98 | 0.94, 0.88-1.00 |
| fPC2 | Late day vs.  early day | 0.93, 0.82-1.04 | 0.92, 0.82-1.03 | 0.92, 0.82-1.04 |
| fPC3 | Early/Late day vs. midday | 0.83, 0.73-0.96 | 0.83, 0.72-0.96 | 0.83, 0.72-0.96 |
| fPC4 | Midday/night vs. early/late day | 0.90, 0.74-1.10 | 0.89, 0.73-1.09 | 0.89, 0.73-1.09 |
| N = 85,728, Cases = 345.  Model 1: Four fPCs and stratified by sex, age group, study region; model 2: model 1 + cardiometabolic disease, height, smoking intensity, alcohol consumption intensity, socio-economic status, education level, sedentary behavior, healthy diet score, hormone therapy, family history of colorectal cancer, and bowel cancer screening; model 3: model 2 + body mass index. fPC: Functional principal component.  Note: Hazard ratios for fPC1 and fPC4 are for a score of +1 vs. 0; for fPC2 and fPC3 for a score of -1 vs. 0 to ease interpretation. | | | | |

# Cox model results after restricting the analysis to never smokers

| **fPC** | **Activity timing** | **Hazard ratio, 95% CI** |
| --- | --- | --- |
| fPC1 | Higher overall vs. lower overall | 0.92, 0.85-0.99 |
| fPC2 | Late day vs. early day | 0.91, 0.79-1.04 |
| fPC3 | Early/Late day vs. midday | 0.87, 0.74-1.02 |
| fPC4 | Midday/night vs. early/late day | 0.96, 0.77-1.20 |
| N = 49,602; Cases = 250.  fPC: Functional principal component; CI: Confidence interval.  Note: Hazard ratios for fPC1 and fPC4 are for a score of +1 vs. 0; for fPC2 and fPC3 for a score of -1 vs. 0 to ease interpretation. | | |

# Cox model results without adjustment for cardiometabolic disease status

| **fPC** | **Activity timing** | **Hazard ratio, 95% CI** |
| --- | --- | --- |
| fPC1 | Higher overall vs. lower overall | 0.94, 0.89-0.99 |
| fPC2 | Late-day vs. early-day | 0.93, 0.85-1.02 |
| fPC3 | Early/late-day vs. mid-day | 0.89, 0.80-0.99 |
| fPC4 | Mid-day/night vs. early/late-day | 1.02, 0.88-1.19 |
| N = 86,252; Cases = 529  fPC: Functional principal component; CI: Confidence interval; model 3 adjustment without adjustment for cardiometabolic diseases  Note: Hazard ratios for fPC1 and fPC4 are for a score of +1 vs. 0; for fPC2 and fPC3 for a score of -1 vs. 0 to ease interpretation. | | |

# Cox model results with adjustment for shift work status

| **fPC** | **Activity timing** | **Hazard ratio, 95% CI** |
| --- | --- | --- |
| fPC1 | Higher overall vs. lower overall | 0.94, 0.89-0.99 |
| fPC2 | Late-day vs. early-day | 0.93, 0.85-1.02 |
| fPC3 | Early/late-day vs. mid-day | 0.89, 0.80-1.00 |
| fPC4 | Mid-day/night vs. early/late-day | 1.02, 0.88-1.20 |
| N = 86,252; Cases = 529  fPC: Functional principal component; CI: Confidence interval; model 3 adjustment + shift work status (1=never, rarely, sometimes, 2=usually, always)  Note: Hazard ratios for fPC1 and fPC4 are for a score of +1 vs. 0; for fPC2 and fPC3 for a score of -1 vs. 0 to ease interpretation. | | |

# Interaction terms for fPCs and covariates

| **P-values of interaction terms** | | | | |
| --- | --- | --- | --- | --- |
| **Covariate** | **fPC1** | **fPC2** | **fCP3** | **fPC4** |
| Sex | 0.2064 | 0.7958 | 0.7173 | 0.9701 |
| Study region | 0.2177 | 0.3881 | 0.9375 | 0.8378 |
| Age group | 0.4709 | 0.6643 | 0.7706 | 0.0557 |
| Height | 0.0531 | 0.6445 | 0.7750 | 0.6674 |
| Body mass index | 0.6440 | 0.8648 | 0.5345 | 0.5283 |
| Townsend Index of Deprivation | 0.3689 | 0.8035 | 0.5433 | 0.5287 |
| Education level | 0.0954 | 0.1304 | 0.8880 | 0.1309 |
| Alcohol use | 0.4758 | 0.3171 | 0.3370 | 0.2214 |
| Pack years of smoking | 0.3409 | 0.7093 | 0.9563 | 0.9346 |
| Diet score | 0.3748 | 0.2445 | 0.8080 | 0.7918 |
| Sedentary behavior | **0.0362** | 0.4671 | 0.6516 | 0.5195 |
| Cardiometabolic diseases | 0.2660 | 0.1670 | 0.1875 | 0.7905 |
| Hormone therapy | **0.0485** | 0.4935 | 0.8997 | 0.9947 |
| History of cancer (mother) | 0.7300 | 0.9526 | 0.8601 | 0.9780 |
| History of cancer (father) | 0.2892 | 0.5041 | 0.6826 | 0.2173 |
| Bowel cancer screening | 0.4685 | 0.9483 | 0.4637 | 0.4862 |

# Sensitivity fPCA with different bandwidth estimations and kernel smoothers

|  | **Fraction of variance explained (%)** | | | | | | | | |
| --- | --- | --- | --- | --- | --- | --- | --- | --- | --- |
|  | *K* | | | | | | | | |
|  | 1 | 2 | 3 | 4 | 5 | 6 | 7 | 8 | 9 |
| **Gaussian kernel** | | | | | | | | | |
| Default | 67.61 | 16.52 | 8.36 | 3.88* | 2.46 | 0.69 | - | - | - |
| GCV | 71.46 | 13.66 | 8.87 | 2.96* | 2.07 | - | - | - | - |
| GMeanGCV | 68.14 | 15.19 | 8.80 | 3.90* | 2.72 | 0.76 | - | - | - |
| **Epanechnikov kernel** | | | | | | | | | |
| Default | 52.17 | 16.21 | 13.61 | 6.63 | 4.07 | 2.90* | 2.03 | 1.02 | 0.93 |
| GCV | 51.10 | 14.97 | 12.96 | 8.95 | 4.80 | 2.73* | 2.03 | 1.13 | 0.86 |
| GMeanGCV | 51.62 | 15.92 | 13.37 | 7.04 | 4.64 | 2.88* | 2.08 | 1.08 | 0.93 |
| *Cumulative fraction of variance explained ≥95%  Note: Default refers to the default settings of the fPCA function in fdapace. GCV is Generalized Cross-Validation; GMeanGCV is Geometric Mean and GCV | | | | | | | | | |

#
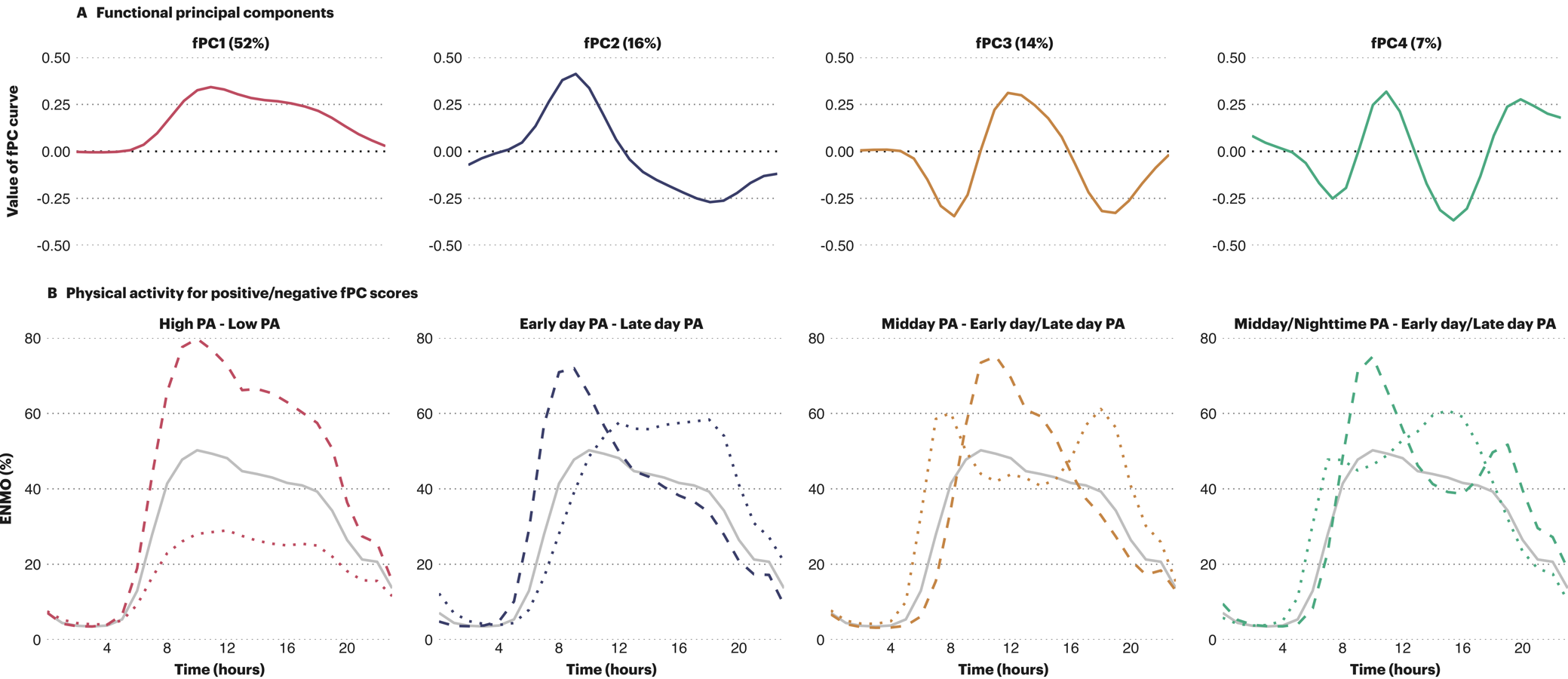
First four fPCs (A) and positive and negative scorers (B) when using an Epanechnikov kernel

# Correlation coefficients for fPCs and derived accelerometry

|  | **Sleep** | **Sedentary behavior** | **Light physical activity** | **Moderate-to-vigorous physical activity** |
| --- | --- | --- | --- | --- |
| **fPC1** | -0.22 | -0.57 | 0.60 | 0.56 |
| **fPC2** | 0.10 | 0.01 | -0.10 | 0.04 |
| **fPC3** | 0.28 | -0.16 | -0.04 | 0.03 |
| **fPC4** | 0.02 | -0.03 | 0.03 | -0.03 |
| Accelerometer-derived variables are the overall average proportion of time spent doing the respective activity. | | | | |
